# Comprehensive analysis of DNA methylation gene expression profiles in GEO dataset reveals biomarkers related to malignant transformation of sinonasal inverted papilloma

**DOI:** 10.1007/s12672-024-00903-7

**Published:** 2024-03-01

**Authors:** Li Mu, Shun Hu, Guoping Li, Ping Wu, Ke Zheng, Sheng Zhang

**Affiliations:** 1 Department of Pathology, The First Affiliated Hospital of Fujian Medical University, Fuzhou, 350005 Fujian China; 2 Department of Pathology, National Regional Medical Center, Binhai Campus of the First Affiliated Hospital, Fujian Medical University, 999 Huashan Road, Fuzhou, 350212 China

**Keywords:** Sinonasal inverted papilloma, Malignant transformation, GEO database, DNA methylation, Tissue microarray, Immunohistochemistry, Biomarkers

## Abstract

**Background:**

DNA methylation may be involved in the regulation of malignant transformation from sinonasal inverted papilloma (SNIP) to squamous cell carcinoma (SCC). The study of gene methylation changes and screening of differentially methylated loci (DMLs) are helpful to predict the possible key genes in the malignant transformation of SNIP-SCC.

**Materials and methods:**

Microarray dataset GSE125399 was downloaded from the Gene Expression Omnibus (GEO) database and differentially methylated loci (DMLs) were analyzed using R language (Limma package). ClusterProfiler R package was used to perform Gene Ontology (GO) analysis on up-methylated genes and draw bubble maps. The Kyoto Encyclopedia of Genes and Genomes (KEGG) pathway and its visualization analysis were analyzed to speculate the possible key Genes in SNIP-SCC malignant transformation. Subsequently, SNIP cases archived in our department were collected, tissue microarray was made, and immunohistochemical staining was performed to analyze the expression levels of UCKL1, GSTT1, HLA-G, MAML2 and NRGN in different grades of sinonasal papilloma tissues.

**Results:**

Analysis of dataset GSE125399 identified 56 DMLs, including 49 upregulated DMLs and 7 downregulated DMLs. Thirty-one genes containing upregulated DNA methylation loci and three genes containing downregulated DNA methylation loci were obtained by methylation microarray annotation analysis. In addition, KEGG pathway visualization analysis of 31 up-methylated genes showed that there were four significantly up-methylated genes including UCKL1, GSTT1, HLA-G and MAML2, and one significantly down-methylated gene NRGN. Subsequently, compared with non-neoplasia nasal epithelial tissues, the expression of HLA-G and NRGN was upregulated in grade I, II, III and IV tissues, while the expression of MAML2 was lost. The protein expression changes of MAML2 and NRGN were significantly negatively correlated with their gene methylation levels.

**Conclusions:**

By analyzing the methylation dataset, we obtained four up-regulated methylation genes UCKL1, GSTT1, HLA-G and MAML2 and one down-regulated gene NRGN. MAML2, a tumor suppressor gene with high methylation modification but loss of protein expression, and NRGN, a tumor gene with low methylation modification but upregulated protein expression, can be used as biological indicators to judge the malignant transformation of SNIP-SCC.

**Supplementary Information:**

The online version contains supplementary material available at 10.1007/s12672-024-00903-7.

## Introduction

Schneiderian papilloma (SP) is a benign tumor with malignant potential originating from ectoderm-derived pseudostratified ciliated columnar epithelium [1], first described by Ward in 1854 and Bilroth in 1855, accounting for about 0.4–4.7% of all nasal and paranasal sinus tumors [2, 3]. According to the WHO classification in 2017, Schneiderian papillomas are classified into three subtypes: Inverted papilloma (IP), Exophytic papilloma (EP), and Oncocytic papilloma (OP) [4]. Among them, IP is more common in men aged 40–70 years, with the highest incidence, accounting for 47–78% of all SPs, followed by EP and OP [5, 6]. IPs usually occurs in the lateral wall of the nasal cavity and maxillary sinus and composed of proliferative epithelium that grows endogenously and thrustingly into the stroma deep below the mucosa. It is composed of squamous and/or respiratory epithelium (ciliated columnar epithelium mixed with mucinous cells) with 5–30 cell layers. The margins are usually smooth and rounded with distinct intact and continuous basement membranes. Neutrophils are commonly infiltrated in the epithelium. The malignant transformation rate of sinonasal inverted papilloma is about 5%-15%, and the common types are squamous cell carcinoma (SCC), verrucous carcinoma and adenocarcinoma [3, 7, 8], but the mechanism is still unclear [9].

Malignant transformation of sinonasal inverted papilloma is a multistep complex process, resulting from the accumulation of multiple genetic and epigenetic changes, often accompanied by continuous molecular abnormalities [10]. Genome-wide DNA methylation analysis was performed on the tissues of inverted sinus papilloma (SNIP) and squamous cell carcinoma derived from the malignant transformation of inverted sinus papilloma (SNIP-SCC), and 11,201 differentially methylated loci (DML) were found in the SNIP-SCC samples [11]. Compared with SNIP, the levels of mature miR-661 mRNA and PLEC protein in SNIP-SCC were significantly upregulated, and the level of OPA3 protein was downregulated. These results suggested that the abnormal expression of MIR661, PLEC, and OPA3 genes may be involved in the malignant transformation of SNIP, indicating that the differentially expressed genes are essential for the molecular process of SNIP-SCC. From papilloma/early SCC to late SCC, LINE-1 hypomethylation increases significantly and was associated with occupational exposure and poor prognosis [12]. These results suggested that aberrant DNA methylation can be used as an early biomarker to evaluate the malignant transformation of sinonasal inverted papilloma. However, the differential expression of gene methylation during malignant transformation of sinonasal inverted papilloma is still insufficient.

In order to further analyze the changes of gene methylation during malignant transformation of sinonasal inverted papilloma, we downloaded the GSE125399 data set of malignant transformation of sinonasal inverted papilloma into squamous cell carcinoma from the GEO (Gene Expression Omnibus) database. DNA DMLs were screened to predict the possible key genes. The R package clusterProfiler was used to analyze the biological functions of the selected DNA methylation loci. Immunohistochemical staining was performed to verify the selected key genes. Combined with histomorphology, the role of these genes in the malignant transformation of sinonasal inverted papilloma was analyzed, as well as the possible key markers and their regulatory networks.

## Materials and methods

### Microarray data

GSE125399 is a gene methylation dataset for malignant transformation of sinonasal inverted papilloma into squamous cell carcinoma (GSE125399 is a methylation profiling by array uploaded by Beijing Tongren Hospital, https://www.ncbi.nlm.nih.gov/geo/query/acc.cgi?acc=GSE125399). GPL13534 platform (Illumina Human Methylation 450 BeadChip) was used for gene methylation sequencing in this dataset, which included 6 SNIPs and 5 SCCs with malignant transformation from SNIP. DNA methylation and gene expression were compared between SNIP and SNIP-SCC.

### Data preprocessing

Data normalization was performed using the normalize arrays function of the Limma software package. We drew boxplots, density distribution map, UMAP maps, and sample distance relationship matrix heat map to observe the distribution of data and samples. We found that one of the samples (GSM3573371) was far away from the other samples in the same group and judged it as an outlier. After the outlier sample was deleted, 5 SNIP and 5 SCC (SNIP-SCC) samples were retained, and the data were preprocessed again. We drew boxplots, density distribution map, UMAP maps, and sample distance relationship matrix heat map, and observed that the filtered data and samples met the requirements.

### Analysis of methylation difference loci

The Limma package was used to analyze the difference of methylation loci, and the volcano map was drawn to show the log2(SNIP-SCC/SNIP) and − log10(adjusted p-value) values of methylation loci. Heat maps were drawn based on significantly upregulated and downregulated methylation loci (The settings of parameters or threshold levels for up-regulation or down-regulation of differentially methylated loci are shown in the Additional file 1: Fig. S2).

### DMLs analysis

The annotated information list of significantly upregulated and downregulated methylation loci was obtained by using the annotated file of the methylation chip. Deletion of duplicated genes resulted in 3 down-methylated genes and 31 up-methylated genes. Two non-coding RNAs (LOC100287834 and LOC391322) were removed from three down-methylated genes, resulting in one down-methylated gene (NRGN).

### GO and KEGG pathway enrichment analysis

We applied clusterProfiler software package to perform GO functional annotation and KEGG pathway enrichment analysis for genes with significantly upregulated DMLs and drew bubble plots to visualize the enriched GO and KEGG results. Two most significant KEGG pathways were found: the drug metabolism pathway and human papillomavirus infection pathway.

### Validation of human SNIP and SNIP-SCC tissue by immunohistochemical staining

Archived cases of SNIP and SNIP-SCC diagnosed in the Department of Pathology of the First Affiliated Hospital of Fujian Medical University from January 2014 to December 2021 were collected. A total of 115 cases were enrolled, including 92 males and 23 females, with an average age of 54.7 ± 11.7 years (range, 28–79 years), including 20 grade I, 63 grade II, 19 grade III and 13 grade IV, and 23 non-neoplastic respiratory epithelial tissues as control group. A 12 × 8 tissue chip was made. The samples involved were validated by the Ethics Committee on Human Research of the First Affiliated Hospital of Fujian Medical University (Approval No. MRCTA, ECFAH of FMU [2022] 389) and all patients provided written informed consent.

### Immunohistochemistry

Antigen repair and immunohistochemical staining of UCKL1 (rabbit polyclonal, 1:2000), GSTT1 (rabbit polyclonal, 1:1500), HLA-G (rabbit polyclonal, 1:300), MAML2 (rabbit polyclonal, 1:2000), and NRGN (rabbit polyclonal, 1:300) were performed according to the instruction manual (KIT-5005 MaxVision™ HRP-Polymer Anti-Rabbit IHC KIT).

### Criteria for staining results

The positive IHC staining of UCKL1, GSTT1, HLA-G, and NRGN was mainly located in the cytoplasm and was scored according to the degree of cytoplasm staining. The degree of staining was divided into 4 grades: 0 for non-staining was negative, 1 for light staining, weakly, 2 for moderate staining, moderately, and 3 for deep staining, strongly expressed.

Immunohistochemical staining of MAML2 was mainly localized in the nucleus. The percentage of positive cells with nuclear staining was counted. Percentage of positive cells < 25% were lost expression, 26–75% were partial expression, and positive cells > 75% indicated complete expression.

### Statistical analysis

The data of each group were first tested for normality, and analysis of variance was used for comparison among multiple groups. P < 0.05 was considered statistically significant. All data were analyzed by SPSS 22.0 statistical software.

## Results

### Normalization of data

**Fig. 1:**
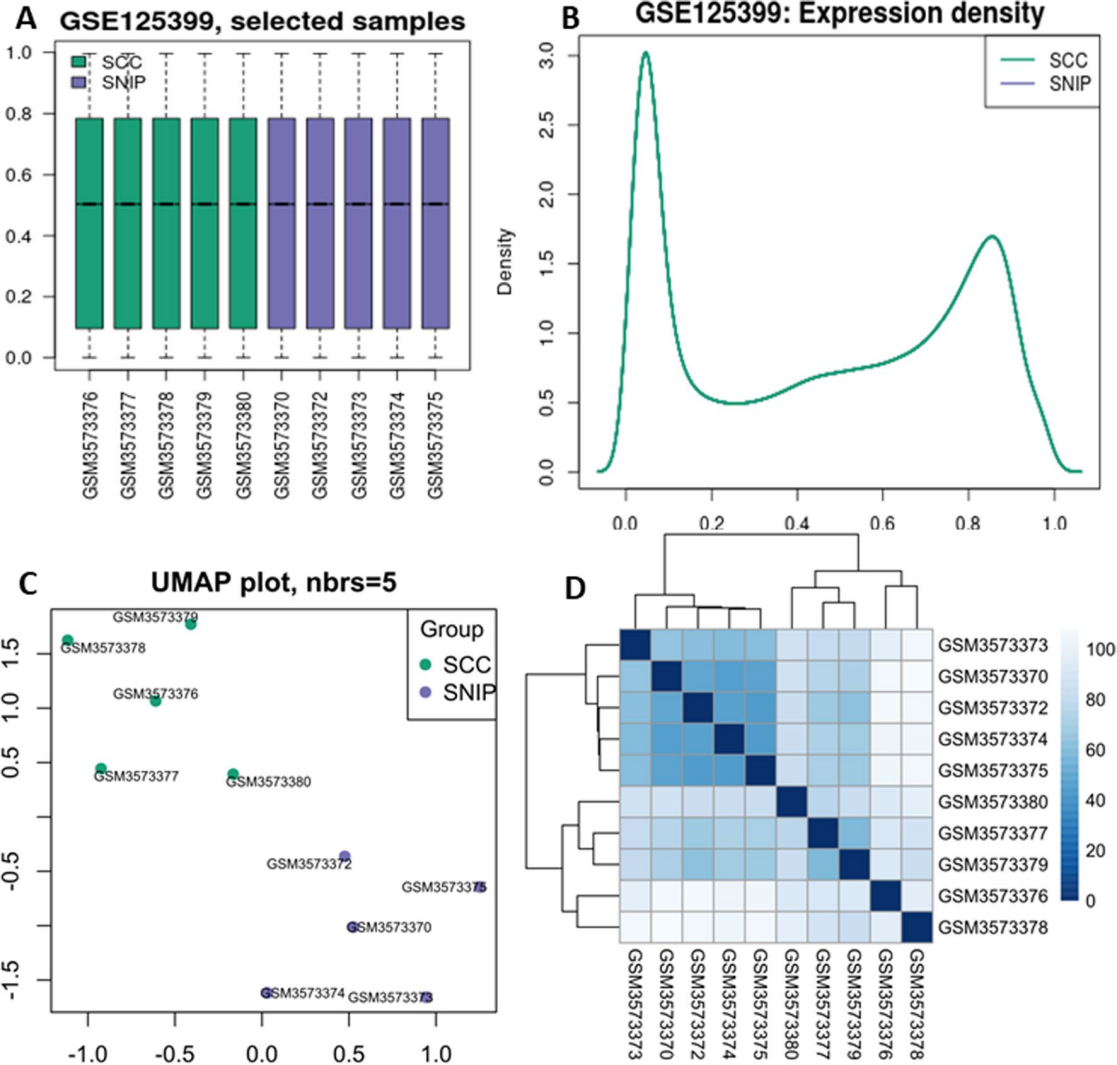
Methylation and sample distribution after deletion of outlier sample and restandardization of data. **A** Boxplot of gene methylation after normalization. Abscissa for individual samples and ordinate for methylation signal values. Green boxed samples are SNIP-SCC group samples and the purple boxed samples are SNIP group samples. **B** Methylation signal density profiles. The abscissa is the methylation signal value and the ordinate is the sample density corresponding to the size of the methylation signal value. Green curves are SNIP-SCC group samples and the purple curves are SNIP group samples. **C** Dimensionality reduction analysis, UMAP plot. The abscissa is the relative distance. Green dots are SNIP-SCC group samples and purple dots are SNIP group samples. The distance between points represents the similarity between samples. **D** Sample distance relationship matrix Heatmap. Rows by columns as individual samples. The color inside the square is proportional to the sample distance, with the closer the distance, the darker the color. Above vs. left are the phylogenetic trees that were hierarchically clustered according to the sample distance, with the closer the distance, the closer the branch distance

After normalized processing of the downloaded dataset GSE125399, it was found that sample GSM3573371 showed outlier performance (Additional file 1: Fig. S1). After removing sample GSM3573371, the boxplot (Fig. 1A), density distribution (Fig. 1B), UMAP (Fig. 1C), and heat map of the sample distance relationship matrix (Fig. 1D) were redrawn. The results showed that the normalized samples had better stability, SNIP and SNIP-SCC datasets presented two independent populations, and the gene expression was different, suggesting that the normalized dataset was more reliable than the original dataset. Normalized data of 5 SNIP and 5 SNIP-SCC samples were obtained, and 481,277 methylation loci were identified.

### Screening of methylation differential loci

Through methylation loci differential analysis, we obtained 56 methylation differential loci (Additional file 1: Fig. S2), including 7 significantly downregulated (cg27518014, cg10989862, cg26069044, cg02185007, et al.) and 49 significantly upregulated (cg14103680, cg07393736, cg01163842, cg11595545, et al.) methylation loci, which can better help distinguish SNIP and SCC samples.

### Screening of differentially methylated genes

**Fig. 2:**
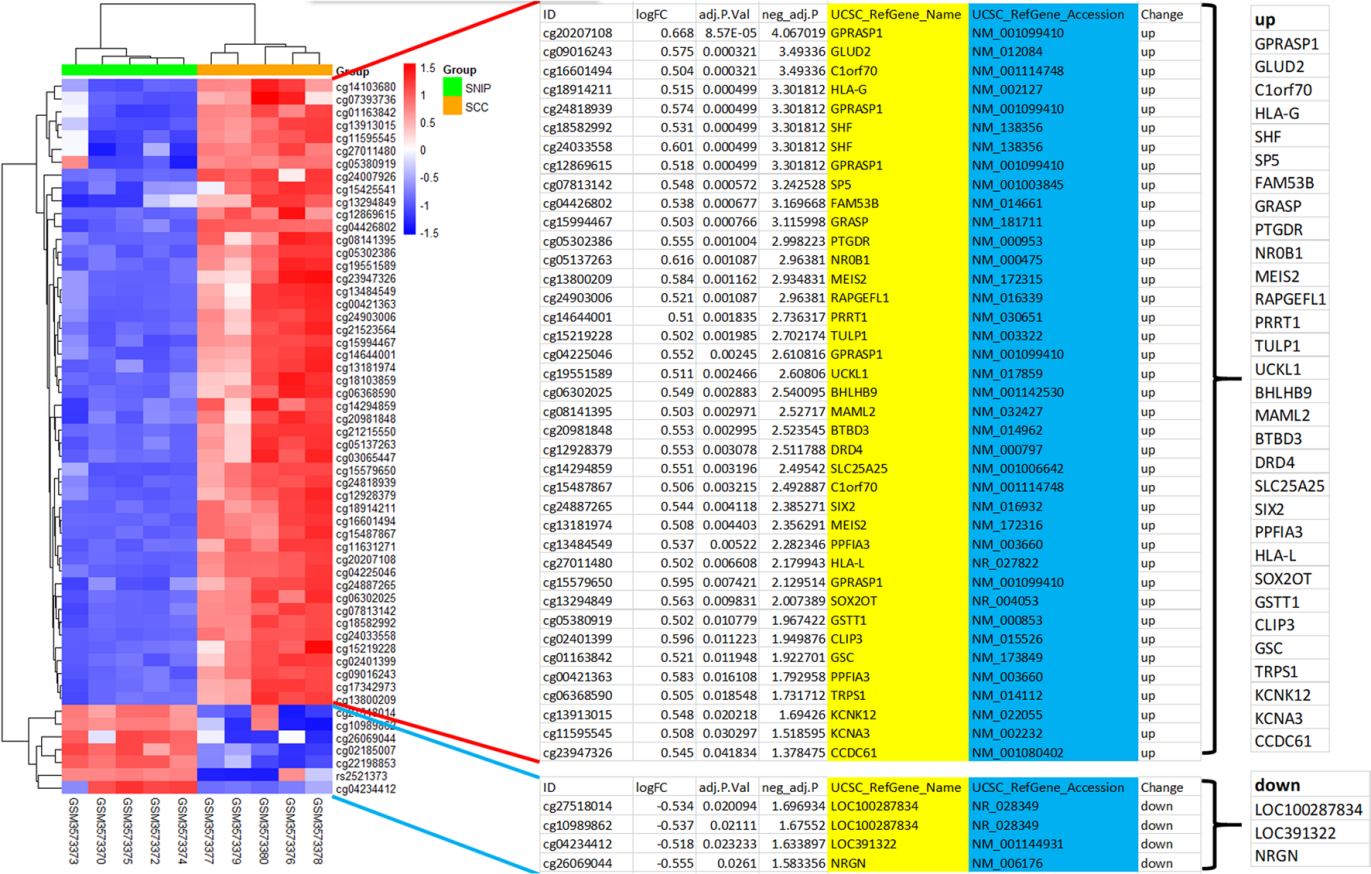
Screening of differentially methylated genes. **A** Methylation loci Heatmap. **B** Methylation loci details. From the left only each column is the probe number, log2 transformed value of fold of methylation signal value, corrected p-value, corrected p-value—log10 transformed value, gene name corresponding to the probe, gene number. **C** Methylated up—versus downregulated genes are displayed, respectively

Thirty-one up-methylated genes (GPRASP1, GLUD2, TMEM240 C1orf70, HLA-G, SHF, SPS, FAM53B, GRASP, PTGDR, NR0B1, MEIS2, RAPGEFL1, PRRT1, TULP1, UCKL1, BHLHB9, MAML2, BTBD3, DRD4, SLC25A25, SIX2, PPFIA3, HLA-L, SOX2OT, GSTT1, CLIP3, GSC, TRPS1, KCNK12, KCNA3 and CCDC61) and three down-methylated genes (Two non-coding RNAs ((LOC100287834 and LOC391322) and one protein-coding gene (neurogranin, NRGN)) were obtained from differentially methylated loci (Fig. 2).

### GO and KEGG pathway enrichment analysis

**Fig. 3:**
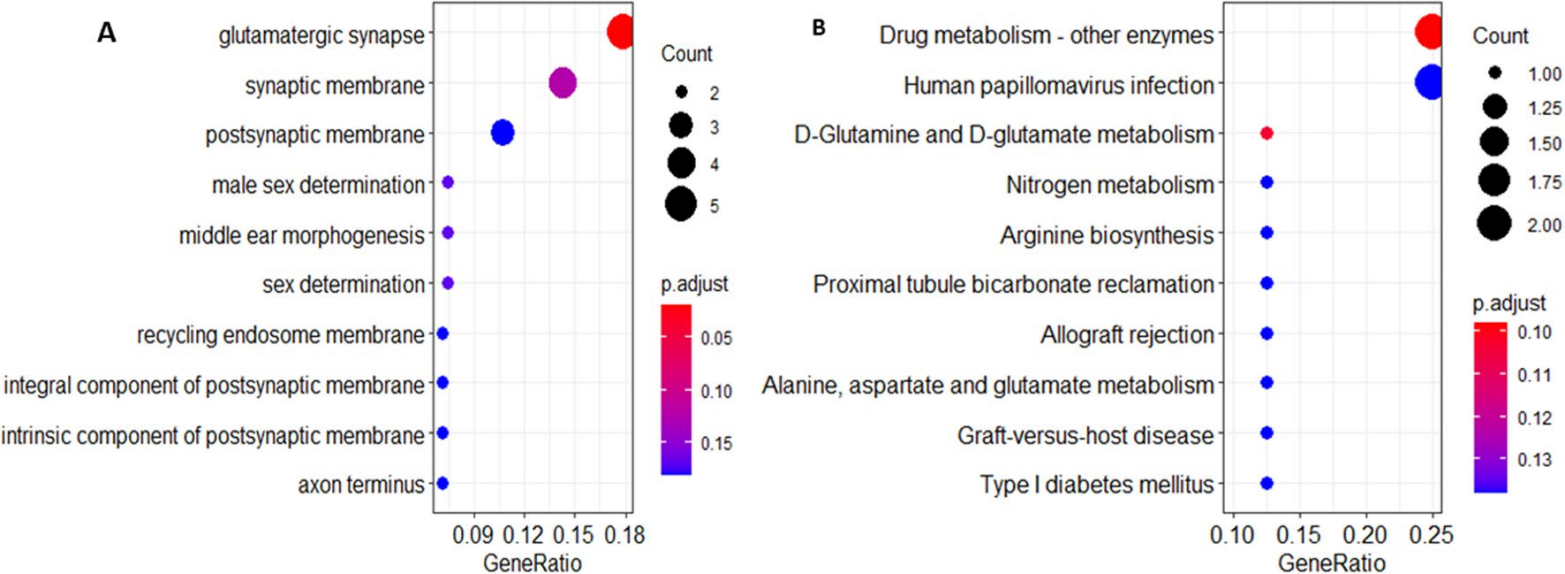
Functional enrichment bubble map of genes with significantly upregulated methylation. **A** Go entry enrichment bubble plot. Abscissa is the proportion of genes identified and the ordinate is the go entry name. **B** KEGG pathway enrichment bubble plot. The abscissa is the proportion of genes identified and the ordinate is the KEGG pathway name. Vs. B, the size of the bubble is proportional to the number of genes identified. The color of the bubble correlates with the corrected P, with smaller P values indicating a more biased red color and vice versa

Figure 3 shows that the three significantly enriched GO items are glutamatergic synapse, synaptic membrane, and postsynaptic membrane (Fig. 3A). The two KEGG pathways that were significantly enriched were Drug metabolism and Human papillomavirus infection (Fig. 3B).

### KEGG pathway visualization analysis

**Fig. 4:**
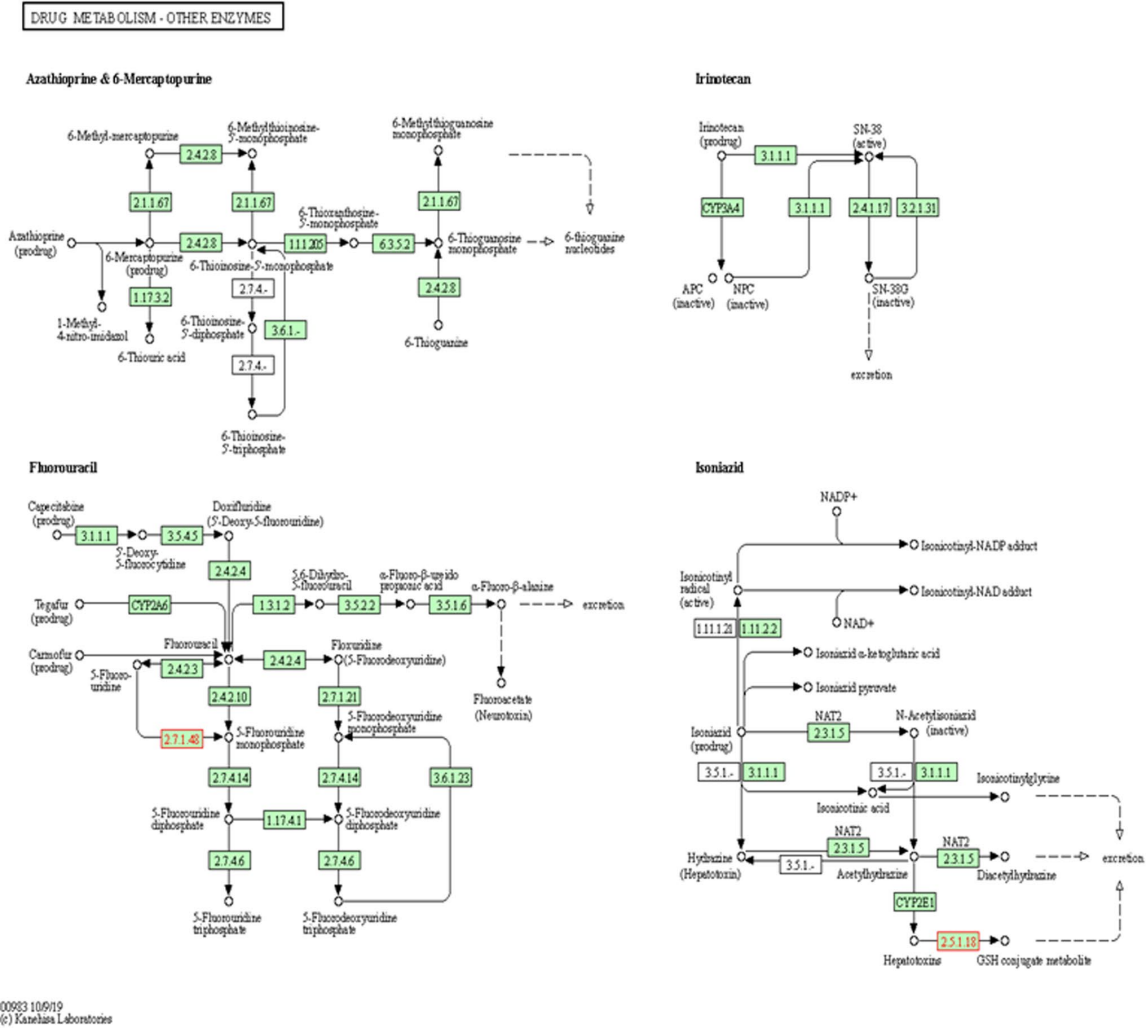
Visualization of drug metabolism pathway. Boxes in the plots represent genes, small circles represent metabolites, lines represent responses, and arrows indicate promotion. Genes in red were among the significantly upregulated genes with methylation identified at 2.7.1.48 (UCKL1) and 2.5.1.18 (GSTT1)

**Fig. 5:**
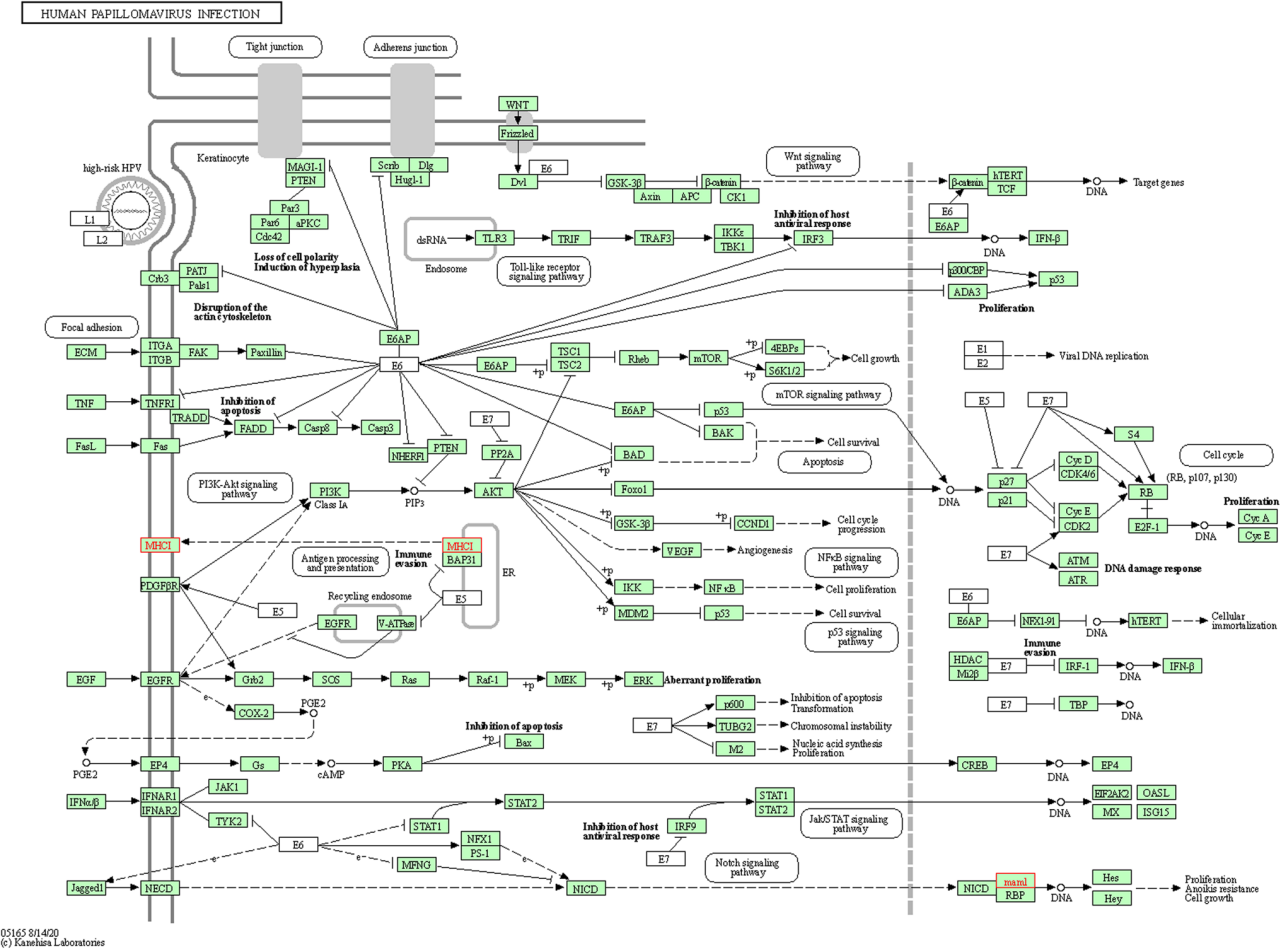
Visualization of human papillomavirus infection pathway. Boxes in the plots represent genes, small circles represent metabolites, lines represent responses, and arrows indicate promotion. Genes with significantly upregulated methylation identified in red are MHCI (HLA-G) and MAML (MAML2)

Two genes, UCKL1 and GSTT1, were significantly upregulated in Drug Metabolism pathway. Two genes, HLA-G and MAML2, were also significantly upregulated in the Human papillomavirus infection pathway. Combined with Figs. 4 and 5, it is speculated that the significant upregulation of UCKL1, GSTT1, HLA-G, and MAML2 methylation and the significant downregulation of NRGN methylation may be the key genes of malignant transformation of sinonasal papillomas.

### Validation of genes with significantly upregulated and downregulated methylation

#### Results of immunohistochemical staining

**Fig. 6:**
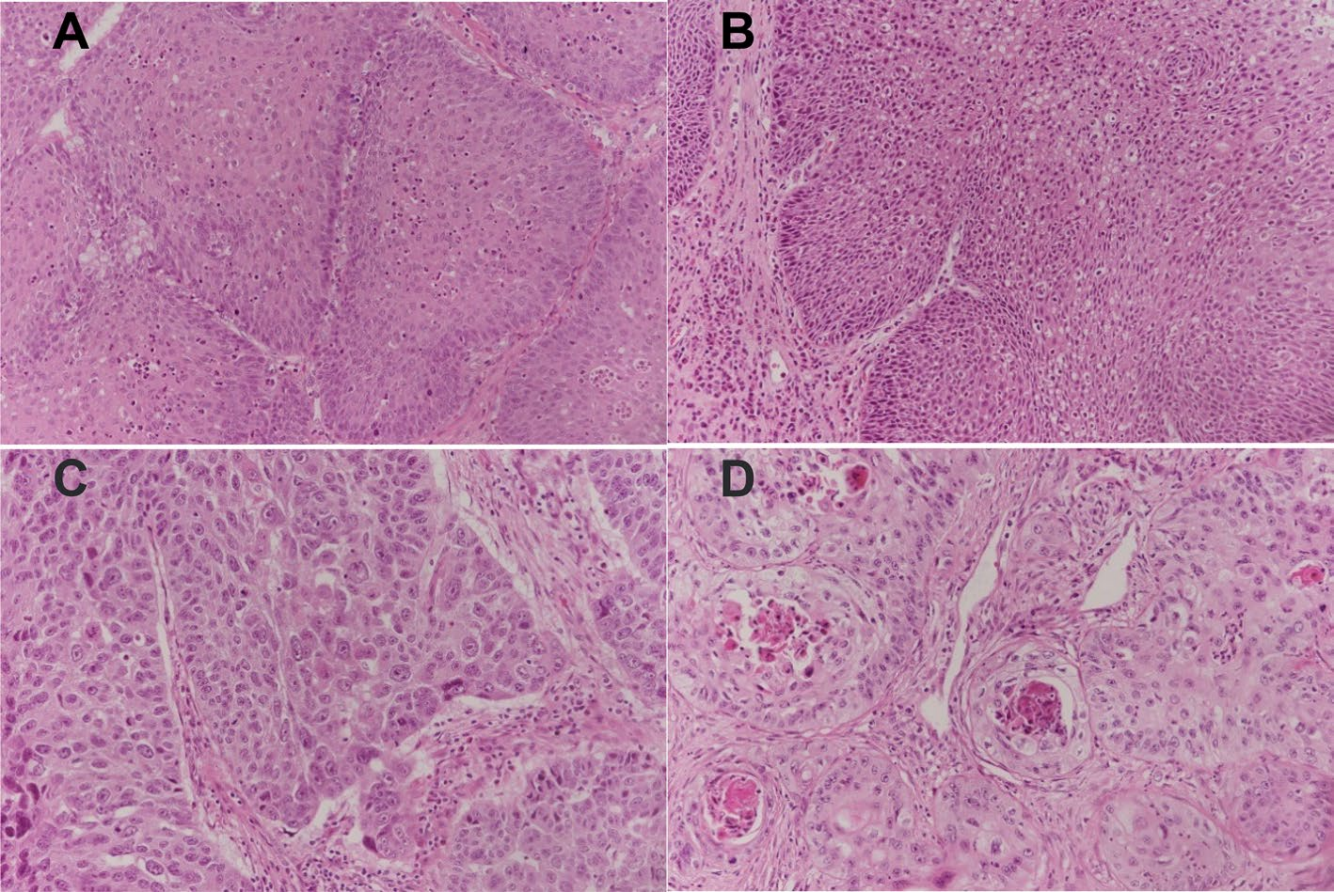
Inverted papilloma histopathology (HE, × 200). **A** Inverted papilloma, Grade I: squamous metaplasia with more than 12 layers of cells, associated with early inversion of squamous metaplasia. **B** Inverted papilloma, Grade II: squamous metaplasia with marked inverted growth and mild cellular atypia. **C** Inverted papilloma, Grade III: almost complete absence of respiratory epithelium, replaced by metaplasia of stratified squamous epithelium, with varying degrees of dysplasia. **D** Inverted papilloma, Grade IV: Invasive SCC was present in the presence of inverted squamous metaplasia and dysplasia, and grade II and III features were present in the diagnosis of grade IV

**Fig. 7:**
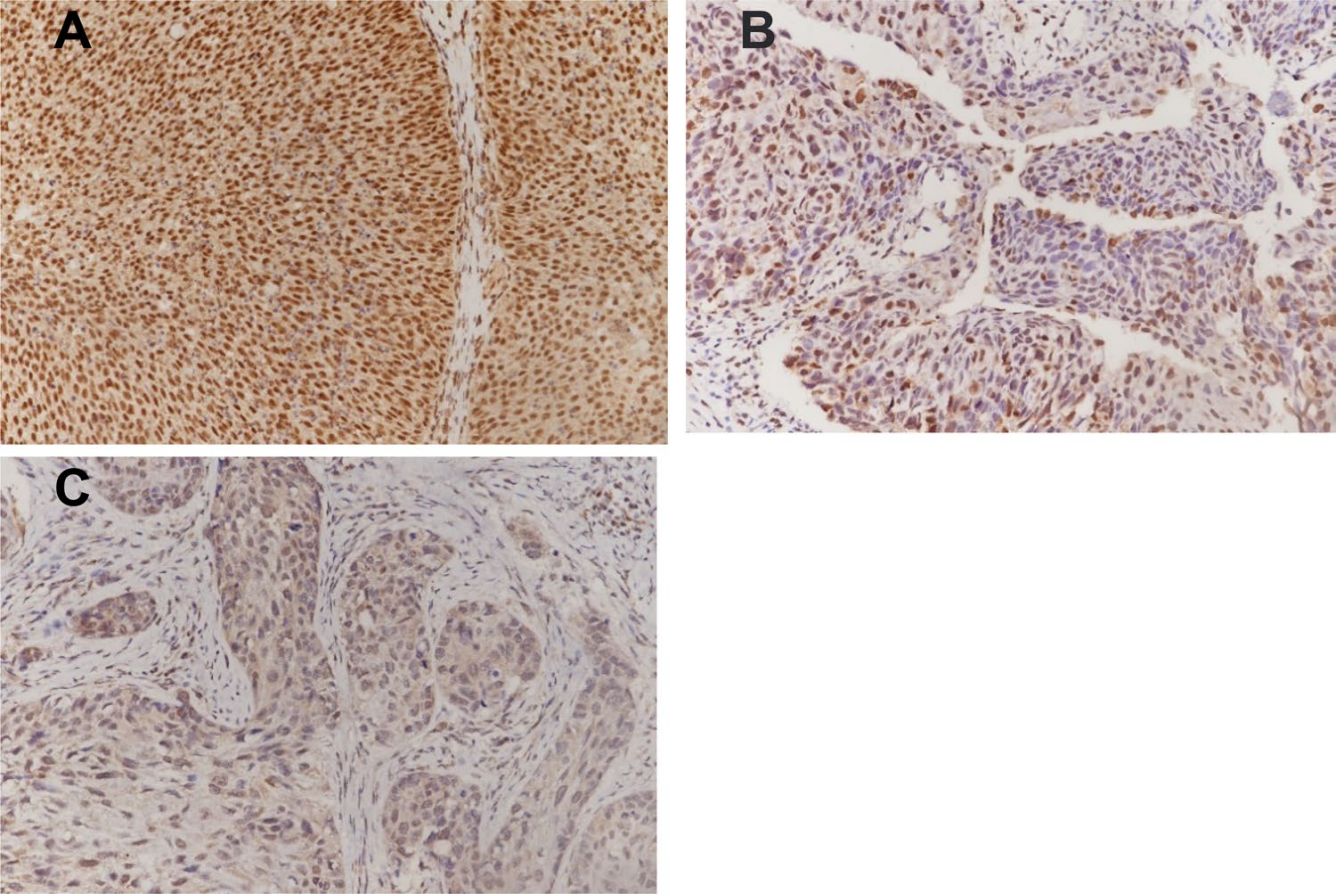
Nuclear expression of MAML2 in sinonasal papilloma (IHC, DAB, × 200). **A** Positive expression in inverted papilloma, Grade I; **B** Partial loss of expression in inverted papilloma, Grade III; **C** Loss of expression in inverted papilloma, Grade IV

Additional file 1: Table S1 and Fig. 6 show the clinicopathological features of inverted papilloma. Figure 7 and Table 1 show that the composition ratio of MAML2 expression loss is different. The loss of MAML2 expression in grade III and IV papillomas is higher than that in other grades, showing a progressive expression loss change.

**Table 1:** Expression of MAML2 in sinonasal papilloma (nuclear expression)

Grade	n	Fully-expression (n, %)	Partial-loss (n, %)	Lost-expression (n, %)
Normal mucosa	23	22 (95.65)	1 (4.35)	0(0)
I	20	16 (80)	4 (20)	0(0)
II	63	37 (58.73)	21 (33.33)	5 (7.94)**
III	19	7 (36.84)	5 (26.32)	7 (36.84)**^##&&^
IV	13	4 (30.77)	4 (30.77)	5 (38.46)**^##&&^

^**^P < 0.01 compared with normal; ^##^*P* < 0.01 compared with I; ^&&^*P* < 0.01 compared with II

Additional file 1: Figs. S3 and S4 and Tables S2 and S3 show no statistically significant differences in the expression of GSTT1 and UCKL1 in grade I, II, III, and IV inverted papilloma (P > 0.05), suggesting that the expression of GSTT1 and UCKL1 may not be related to the deterioration of SNIP-SCC.

Additional file 1: Fig. S5 and Table S4 show that there was no significant difference in HLA-G expression between inverted papilloma and normal nasal mucosa (P > 0.05); However, with the progression of sinonasal papilloma and the onset of malignant transformation, the expression of HLA-G increases, which may be unrelated to HLA-G hypermethylation.

**Fig. 8:**
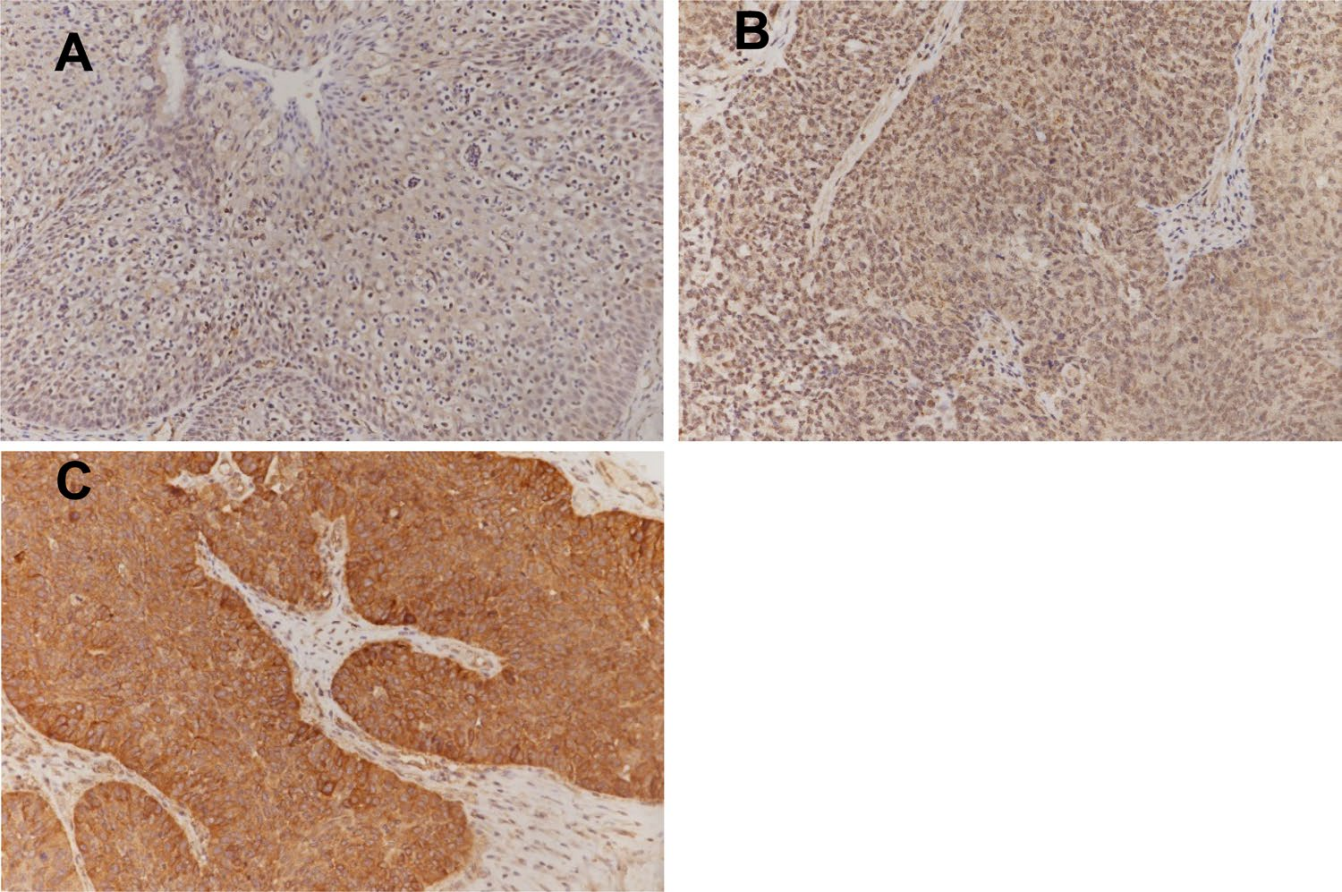
Expression of NRGN in sinonasal papilloma (IHC, DAB, × 200). **A** Weak expression of NRGN in inverted papilloma, GradeI; **B** Moderate expression of NRGN in inverted papilloma, Grade II; **C** High expression of NRGN in inverted papilloma, Grade IV

Figure 8 and Table 2 show that the expression of NRGN in inverted papilloma is gradually upregulated in a rank-dependent manner, and the expression level is significantly negatively correlated with methylation level. The degree of NRGN expression is involved in the process of malignant transformation.

**Table 2:** NRGN expression (Cytoplasmic expression) in sinonasal papillomas

Grade	n	Negative (n, %)	Weak-Expression (n, %)	Medium-Expression (n, %)	Strong-Expression (n, %)
Normal mucosa	23	0 (0)	16 (69.57%)	7 (30.43%)	0 (0)
I	20	0 (0)	6 (30%)	13 (65%)	1 (5%)*
II	63	4 (6.35%)	19 (30.16%)	37 (58.73%)	3 (4.76%)**
III	19	1 (5.26%)	1 (5.26%)	11 (57.89%)	6 (31.58%)**^#&^
IV	13	1 (7.69%)	1 (7.69%)	7 (53.85%)	4 (30.77%)**^##&^

^**^P < 0.01 compared with normal; #P < 0.05, ## P < 0.01 compared with Grade I; &P < 0.05 compared with Grade II

#### Relationship between gene methylation level and immunohistochemical staining results

Compared with the results of bioinformatics analysis and immunohistochemical staining, we found that with the progression of sinonasal papilloma, the expression of MAML2 protein decreased or even lost was negatively correlated with the significantly upregulated gene methylation and the increased expression of NRGN was negatively correlated with the significant downregulation of gene methylation, which were in line with the general rule of the relationship between gene methylation and protein expression. These results suggest that MAML2 and NRGN may be the key genes involved in DNA methylation during SNP-SCC deterioration, and MAML2 can be used as a predictor of malignant transformation of sinonasal papilloma.

### Analysis of key methylated gene interaction network

**Fig. 9:**
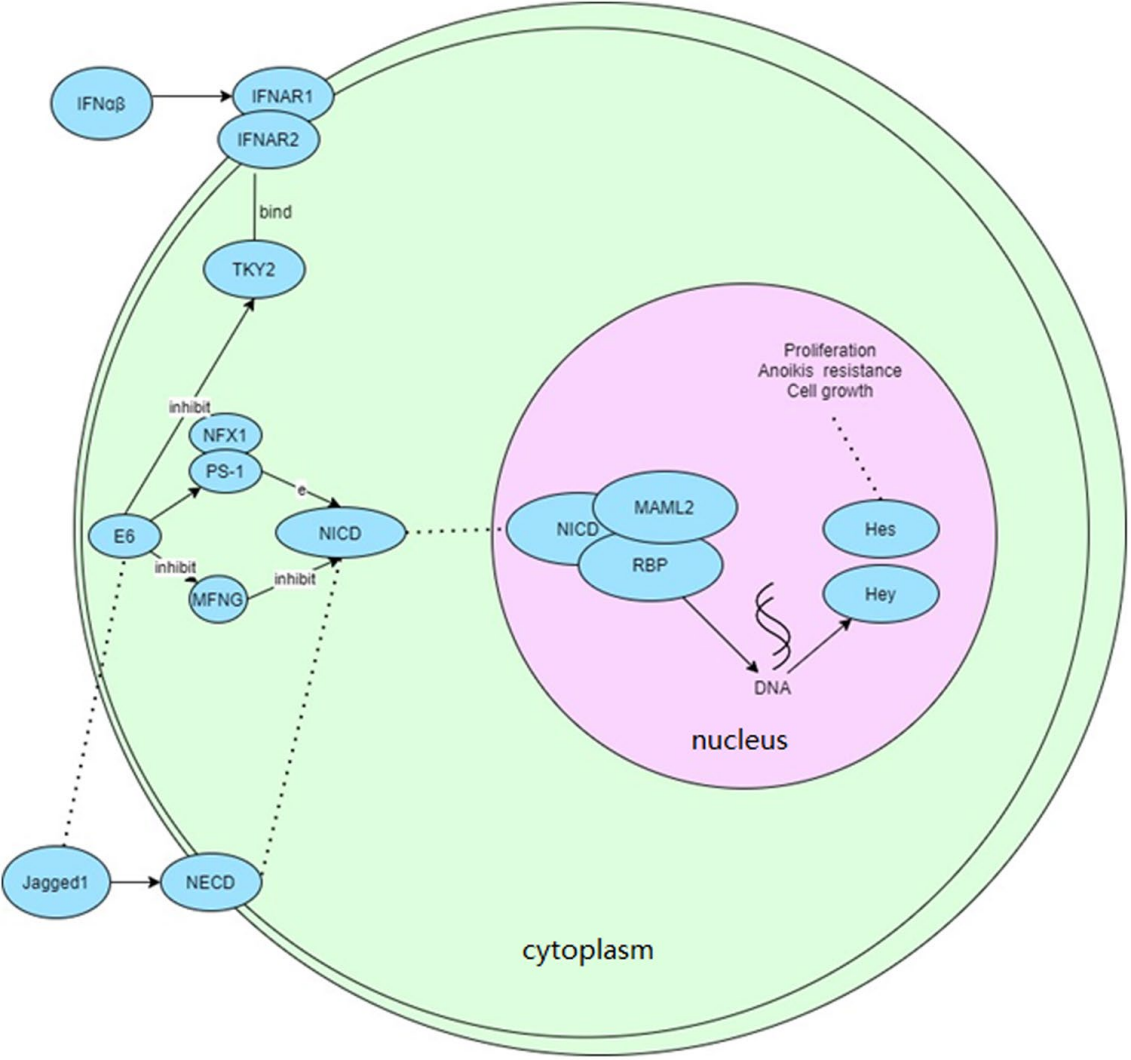
MAML2 related pathways. IFNAR1/IFNAR2 on the cell membrane binds to IFNαβ outside the cell membrane and then binds to TKY2. E6 is a key point. E6 could inhibit TKY2 and promote the binding of PS-1 and NFX1, and then act on NICD through enzymes. Alternatively, E6 could inhibit NICD by inhibiting MFNG expression. NICD acts indirectly on the NICD/MAML2/RBP complex in the nucleus. Jagged1 promotes NECD expression on the cell membrane and acts indirectly on NICD, thereby indirectly regulating the NICD/MAML2/RBP complex in the nucleus. In the nucleus, MAML2 forms a complex with NICD and RBP. NICD/MAML2/RBP acts on DNA, and DNA acts on Hes and Hey to proliferate/inhibit Anoikls/ promote cell growth. MAML2 was an activator of the NOTCH pathway. The DNA methylation profile of the entire MAML2 promoter and gene body in cancer cells was characteristic of active genes. Loss of MAML2 expression reflected malignant transformation of sinonasal papilloma

Figure 9 shows the NOTCH signaling pathway during malignant transformation of sinonasal papilloma obtained by bioinformatics analysis of GSE125399 dataset. Our validation results reconfirmed that MAML2 is an activator of the NOTCH pathway, the DNA methylation profile of the entire MAML2 promoter and gene body in cancer cells is characteristic of active genes, and the loss of MAML2 expression is the embodiment of malignant transformation of sinonasal papilloma.

**Fig. 10:**
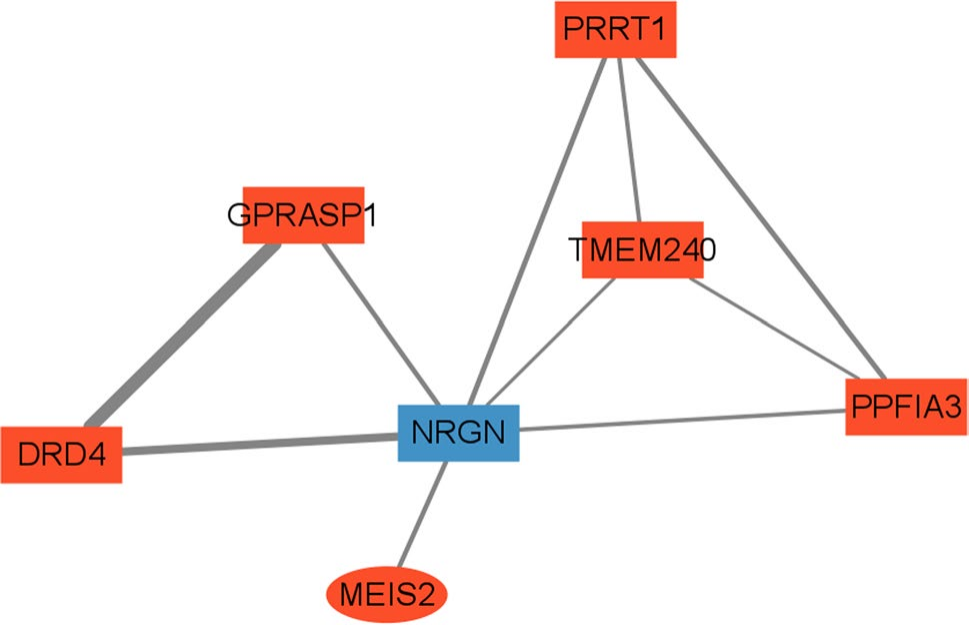
Interaction network diagram with NRGN as the center. The dots are molecules, and the up-methylated genes are shown in red and the down-methylated genes are shown in blue. Ellipses are transcription factors and squares are other types of molecules. The line between nodes is the interaction between molecules. The thickness of the line indicates the strength of the interaction relationship. The stronger the interaction, the thicker the line

The molecular action network diagram of NRGN gene was not found in KEGG database. The action network diagram of NRGN was found in STRING database and TFCheck database. Figure 10 shows that NRGN directly interacts with DRD4, GPRASP1, MEIS2, PRRT1, TMEM240, and PPFIA3 to regulate cell proliferation.

## Discussion

The Gene Expression Omnibus (GEO) includes high-throughput Gene Expression data submitted by researchers around the world, including memory chips, next-generation sequencing, and other single-cell sequencing data [13, 14]. Wu et al. downloaded five DNA methylation and gene expression profiling datasets (GSE60185, GSE42568, GSE21653, GSE58812 and GSE52865) from GEO database and identified lots of differentially expressed genes and abnormally methylated genes between breast cancer samples and normal samples. The relationship between DMLs and the prognosis of breast cancer was determined, suggesting that these genes could be used as prognostic and diagnostic markers of breast cancer [15]. DNA methylation represents a new and very promising area of research. Many key tumor suppressor genes, which are silenced by DNA methylation, have been evaluated for their prognostic and predictive significance in many types of cancer [16]. At present, there is only one dataset on malignant transformation of sinonasal papilloma, namely, GSE125399. Illumina Human Methylation 450 BeadChip Methylation analysis was used to study DNA Methylation of CpG islands and promoters in 6 SNIP samples and 5 SNIP-SCC samples. For the first time, differences in DNA methylation between SNIP and SNIP-SCC were compared. Through bioinformatics analysis on the methylation dataset GSE125399, we found that MAML2, UCKL1, GSTT1, and HLA-G methylation significantly upregulated and NRGN methylation significantly downregulated may be the key genes of sinonasal papilloma malignant transformation.

In order to reduce the systematic errors and improve the repeatability of the study, we need to optimize the missing replacement of the downloaded methylation data, reduce the batch error effect, and eliminate the outlier samples. The results showed that the processed samples had better stability, SNIP and SNIP-SCC datasets showed two independent populations, and the gene expression showed differences. The normalized data is conducive to further data analysis.

As one of the biomarkers related to cancer, the identification and application of DNA differential methylation patterns is one of the research hotspots. In 75 colorectal cancer samples, 4062 methylation loci were detected, of which 26 promoter hypermethylation genes and 143 promoter CpG hypomethylation genes were found, suggesting that these DNA methylation biomarkers may be important diagnostic markers and therapeutic targets for colorectal cancer [17]. The methylation status of 485,577 CpG loci in 24 gallbladder carcinoma tissues (tumor, adjacent non-tumor tissue and 8 gallstones each) was studied, of which 24,188 (72%) were hypermethylated and 9255 (28%) were hypomethylated. A comparative analysis of differential proteomic data revealed 7 hypermethylated or down-regulated genes (e.g., FBN1, LPP, SOD3) and 61 hypomethylated or up-regulated genes (e.g., HBE1, SNRPF, TPD52), which contributed to the early diagnosis of gallbladder cancer [18]. Compared with adenomas, Galamb et al. found that the methylation levels of AXIN2, DKK1, VANGL1, and WNT5A gene promoters in colorectal cancer were higher, while the methylation levels of SOX17, PRICKLE1, DAAM2 and MYC were lower, and subsequently confirmed the negative correlation between expression and methylation [19]. Among the differentially methylated loci in the malignant transformation dataset of sinonasal papilloma, we obtained 31 genes containing up-methylated loci and 3 genes containing down-methylated loci. The 31 genes containing upregulated methylation loci included MAML2, GLUD2, GRASP, UCKL1, CLIP3, GSTT1, HLA-G, etc. The three genes containing downregulated loci included two non-coding RNAs (LOC100287834 and LOC391322) and NRGN. Studies of differentially expressed genes with methylation are most often used to uncover candidate genes associated with tumors. By comparing gene methylation levels in cancer tissues and adjacent normal tissues, active genes, methylation loci, and possible suppressor genes leading to tumorigenesis and development can be found out, thereby laying a foundation for the study of specific markers and molecular targets of tumors. We analyzed GO enrichment and KEGG pathway enrichment of DMLs and found that DMLs were mainly enriched in single-stranded DNA binding, chromatin binding, double-stranded DNA binding, DNA-dependent ATPase activity, drug metabolism and human papillomavirus infection pathway. Evaluation of GO functional enrichment and KEGG pathway analysis of DMLs can help us better understand the SNIP-SCC genetic regulatory network and the regulatory mechanisms involved in biological processes. KEGG pathway visualization showed that UCKL1 and GSTT1 were upregulated in the drug metabolism pathway, and HLA-G and MAML2 were significantly upregulated in the human papillomavirus infection pathway. It is speculated that the significant upregulation of UCKL1, GSTT1, HLA-G, and MAML2 methylation and the significant downregulation of NRGN methylation may be the key genes of malignant transformation of sinonasal papilloma.

Amplification targeted sequencing was used to analyze 409 gene mutations in 6 papilloma/cancer tissue samples from 4 SNIP-derived SCC patients. The results showed that CDKN2A, KMT2D, NF1, PDE4DIP, CYP2D6, FLT4, and MYH9 were mutated in several cases [20]. The results of SCCA1, SCCA2 and SCC antigen analysis in serum and tissue samples from 18 SNIP patients and 23 sinus SCC patients showed that the serum SCCA1 concentration in SNIP patients was significantly higher than that in SCC patients, and the serum SCCA2 level in SCC group was significantly higher than that in SNIP group [21]. Compared with SNIP tissue, downregulation of DLEC1 cilia and flagellate-associated protein (DLEC1) in SCC tissue is associated with promoter hypermethylation [22]. We collected 115 cases of sinonasal papilloma and malignant transformation, determined the expression of aberrant methylated genes by immunohistochemical staining, and performed a comprehensive analysis to identify promising DNA methylation biomarkers for the diagnosis of malignant transformation of sinonasal papilloma. The results showed that MAML2 expression decreased in a hierarchical manner and the expressions of NRGN and HLA-G were increased, while the expressions of GSTT1 and UCKL-1 were not significantly changed during the deterioration of SNIP-SCC. Further comparison of DNA methylation status revealed that HLA-G expression was upregulated and hypermethylated, indicating that its expression changes in SNIP-SCC may not be regulated by DNA methylation, or there are other potential regulatory mechanisms that we have not yet discovered, which need to be further studied.

MAML2, a member of the Mastermind-like protein family, is a coactivator of the oncogenic NOTCH signaling pathway [23] and plays a key role in cell proliferation, metastasis and epithelial-mesenchymal transition [24–26]. MAML2 is abnormally expressed in various cancers [27], for example, MAML2 enhancer region methylation is significantly increased in breast cancer and squamous cell carcinoma [28–30]. MAML2 expression was found to decrease after DNA methylation and histone modification [31]. MAML2 enhancer was hypomethylated and its expression was at a high level in breast cancer [32]. A study based on glioma microarray data identified MAML2 as a novel gene associated with glioma [33]. MAML2 overexpression or MAP3K1 deletion induced meki resistance. Consistent with its function as a transcriptional coregulator of NOTCH, MAML2 overexpression leads to the activation of the NOTCH target HES1. In breast cancer and melanoma, activated NOTCH promotes acquired resistance to MAPK inhibitors [34]. Our immunohistochemical staining results showed that MAML2 expression was gradually lost during the malignant transformation of SNIP-SCC, but its gene was hypermethylated.

UCKL1 is a catalytically active protein with uridine cytidine kinase and phosphoribosyltransferase, which can promote the growth of tumor cells. Serum UCKL-1 mRNA levels were found to be increased 100–1000 times in patients with breast cancer, with the highest UCKL-1 expression in Luminal A and HER2 (ERRB2) subtypes [35]. Studies have found that upregulation of UCKL1 expression may be an indicator of adverse prognosis in hepatocellular carcinomas [36]. Immunohistochemistry was used to evaluate the expression of UCKL-1 in HCC tissues, and the results showed that the expression of UCKL-1 in HCC tissues was significantly elevated [37]. Down-regulating the expression of UCKL-1 in leukemia cells by RNA interference was found to aggravate apoptosis and slow down the cell cycle process, thus reducing the growth rate of leukemia cells with small interference with UCKL-1 RNA processing [38]. In this study, immunohistochemical staining of SNIP and SNIP-SCC cases showed no significant difference in UCKL-1 expression between normal nasal mucosa tissues and grade I, II, III and IV tissues of inverted papilloma.

Glutathione S-transferases (GSTs) belong to the superfamily of phase II metabolic enzymes and play an important anticancer role by catalyzing the binding of glutathione to electrophiles that induce ROS production by heterogenin [39, 40]. The main GST enzymes are GSTM1, GSTT1, and GSTP1, among which the phenotypic loss of GSTT1 activity is due to its homozygous loss. Hypermethylation of CpG islands in the GSTT1 promoter region leads to loss of gene expression and is associated with increased susceptibility of cells to carcinogens [41–43]. Studies have found that GSTT1 gene promoter hypermethylation changes are found in a variety of human tumors [44]. The promoter methylation of the GSTT1 gene was found to be higher in patients with breast cancer than in healthy controls, a risk factor for breast cancer [45]. It was found that GSTT1 expression may be associated with an increased risk of colorectal cancer in Asians [46]. In 750 patients with oral squamous cell carcinoma (OSCC), GSTT1 protein expression was significantly upregulated, and was significantly higher in smoking and drinking patients than in controls, suggesting that GSTT1 and history of smoking and drinking are closely related to OSCC susceptibility. However, no significant differences in GSTT1 protein levels were observed between patients with cervical squamous cell carcinoma and healthy subjects. In this study, GSTT1 expression was not found to be associated with malignant transformation of SNIP-SCC.

Human leukocyte antigen G (HLA-G) is a non-classical MHC class I molecule originally identified in trophoblast cells at the maternal–fetal interface and plays a key role in protecting fetal allogeneic tissues from maternal immune rejection [47]. HLA-G is highly expressed in many solid tumor cells and is positively correlated with infiltrating immune cells in the tumor microenvironment [48–51], which may be a means for tumor cells to avoid immune system regulation by inhibiting natural killer and T cell-mediated lysis [52, 53]. HLA-G is considered as a novel tumor immune checkpoint molecule. Functional enrichment analysis showed that HLA-G was mainly related to T-cell activation, T-cell regulation, and lymphocyte-mediated immunity [54]. The relationship between HLA-G overexpression and aberrant DNA methylation remains elusive. It was found that in human mesenchymal stem cells and placental tissues, the hypomethylation of CpG island occurred not only in the proximal end of the HLA-G promoter, but also in the gene body [55]. The expression of HLA-G was upregulated in breast cancer and malignant melanoma was found, which was partially regulated by DNA methylation [56]. HLA-G was highly expressed in the breast cancer cell line McF-7 and its DNA was hypomethylated [57]. DNA methylation can induce HLA-G overexpression in McF-7 cells, which may be a potential target of new anticancer drugs. Studies have found that high expression of HLA-G is associated with breast cancer metastasis [58]. The expression of HLA-G in oral squamous cell carcinoma was higher than that in normal oral mucosa, which was correlated with tumor stage and lymph node metastasis [59]. However, the expression of HLA-G protein was significantly downregulated in oral squamous cell carcinoma. Transfection of HLA-G overexpression vector could significantly reduce cell viability, migration, and invasion, while inhibition of HLA-G expression could significantly enhance these properties [60]. HLA-G protein was highly expressed in vulvar squamous cell carcinoma, which was correlated with clinical stage, tumor size, and tumor invasion depth, and patients with low HLA-G expression had a good prognosis [61]. The results of this study showed that with the progression and malignant transformation of sinonasal inverted papilloma, the expression of HLA-G increased and was significantly correlated with tumor grade, suggesting that it may be involved in the progression of SNIP-SCC. However, there was no significant difference between the expression of HLA-G in inverted papilloma and that in normal nasal mucosa, suggesting that HLA-G hypermethylation may not be related to the progression of inverted papilloma.

NRGN, a human homolog of the neuron-specific rat RC3/neurogranin gene, exhibits aberrant expression in the brain and plays a role in the development of Parkinson's disease, schizophrenia and Alzheimer's disease [62–64]. Little is known about the role of NRGN in tumors, except that NRGN expression is reduced in gliomas and T-cell lymphomas [65]. The low expression of NRGN in GBM plays an antitumor effect in the progression of glioma, which can be used as a new therapeutic target [66, 67]. Studies have shown that NRGN can be produced at high levels outside the brain and has a novel tumor suppressor effect in murine T-cell lymphoma [61]. Studies have revealed that LINC00641 acts as a ceRNA in glioma cells by upregulating NRGN by absorption of miR-4262. Silencing NRGN expression can counteract the inhibition of glioma cell proliferation caused by upregulation of LINC00641 [67]. RBPMS-AS1 promoted NRGN transcription and enhanced radiosensitivity of GBM through miR-301a-3p/CAMTA1 axis [68]. Immunohistochemical staining showed high expression of DNCH2, ARHGEF6, NPM1, and SRI, while low expression of NRGN and TM4SF2 in gliomas [66]. It is not known whether NRGN is regulated by DNA methylation. Our study found a significantly downregulated methylation loci CG26069044 in the NRGN gene in SNIP-SCC, which may be a new finding of NRGN expression dysregulation in cancer. At the same time, immunohistochemical staining showed that the expression of NRGN was increased in SNIP-SCC, suggesting that the expression of NRGN may be involved in the process of malignant transformation.

In general, we found that the expression of MAML2 was downregulated and the gene was hypermethylated, while the expression of NRGN was upregulated and the gene was hypomethylated. There was a significant negative correlation between the expression levels of MAML2 and NRGN and the methylation status, which further confirmed the stability and reliability of our study results. These results suggest that MAML2 and NRGN may serve as methylation biomarkers and potential therapeutic targets for accurate diagnosis and treatment of SNIP-SCC.

## Conclusions

By analyzing the methylation dataset of sinonasal papilloma and malignant transformation (GSE125399), 31 differentially methylated upregulated genes and 3 down-methylated genes were identified. GO functional enrichment and KEGG pathway analysis revealed that these genes were mainly enriched in glutamatergic synapses, synaptic membranes and postsynaptic membranes, drug metabolism, and human papillomavirus infection pathways. KEGG pathway visualization analysis revealed four up-methylated genes, UCKL1, GSTT1, HLA-G, and MAML2, and one significantly down-methylated gene NRGN. The results of tissue microarray and immunohistochemical staining showed that the expression of HLA-G and NRGN was upregulated and the expression of MAML2 was downregulated in the process of SNIP-SCC deterioration. The protein expression of two key genes was opposite to the methylation level, namely, the hypermethylation of MAML2 resulted in its expression downregulation, and the hypomethylation of NRGN resulted in its expression upregulation. Therefore, we hypothesized that MAML2 tumor suppressor gene and NRGN oncogene might be potential prognostic biomarkers and therapeutic targets of SNIP-SCC.

### Supplementary Information

**Additional file 1: Table S1.** Clinicopathological parameters of sinonasal papillomas. **Table S2.** GSTT1 expression (Cytoplasmic expression) in sinonasal papillomas. **Table S3.** UCLK1 expression (Cytoplasmic expression) in sinonasal papillomas. **Table S4.** HLA-G expression (Cytoplasmic expression) in sinonasal papillomas. **Figure S1.** Methylation and sample distribution before data normalization. **Figure S2.** Screening of differentially methylated loci. **Figure S3.** Expression of GSTT1 in sinonasal papilloma (IHC, DAB, ×200). **Figure S4.** Expression of UCLK1 in sinonasal papilloma (IHC, DAB, ×200). **Figure S5.** Expression of HLA-G in sinonasal papilloma (IHC, DAB, ×200).

## Data Availability

The datasets used in this study are from free public resources, GEO (https://www.ncbi.nlm.nih.gov/geo/) repository (https://www.ncbi.nlm.nih.gov/geo/) repository. [https://www.ncbi.nlm.nih.gov/geo/query/acc.cgi?acc=GSE125399].
