# Supplementary material for: Comprehensive analysis of DNA methylation gene expression profiles in GEO dataset reveals biomarkers related to malignant transformation of sinonasal inverted papilloma
Source: Discov Oncol. 2024 Mar 1;15:53. doi: 10.1007/s12672-024-00903-7 (PMC10907326; doi:10.1007/s12672-024-00903-7)
Supplement: Supplementary file 1 — Additional file 1: Table S1. Clinicopathological parameters of sinonasal papillomas. Table S2. GSTT1 expression (Cytoplasmic expression) in sinonasal papillomas. Table S3. UCLK1 expression (Cytoplasmic expression) in sinonasal papillomas. Table S4. HLA-G expression (Cytoplasmic expression) in sinonasal papillomas. Figure S1. Methylation and sample distribution before data normalization. Figure S2. Screening of differentially methylated loci. Figure S3. Expression of GSTT1 in sinonasal papilloma (IHC, DAB, ×200). Figure S4. Expression of UCLK1 in sinonasal papilloma (IHC, DAB, ×200). Figure S5. Expression of HLA-G in sinonasal papilloma (IHC, DAB, ×200). [file 12672_2024_903_MOESM1_ESM.docx]

**Additional Materials**

Comprehensive Analysis of DNA Methylation Gene Expression Profiles in GEO Dataset Reveals Biomarkers Related to Malignant Transformation of Sinonasal Inverted Papilloma

Li Mu, Shun Hu, Guoping Li, Ping Wu, Ke Zheng*, Sheng Zhang*

Department of Pathology, The First Affiliated Hospital of Fujian Medical University, Fuzhou, Fujian, 350005, China

Li Mu: 1362013589@qq.com

Shun Hu: 69166963@qq.com

Guoping Li: liguoping_827@126.com

Ping Wu: 99629286@qq.com

**Corresponding Author:** Ke Zheng* ([keerzheng@fjmu.edu.cn](mailto:keerzheng@fjmu.edu.cn)); Sheng Zhang (zhgshg@fjmu.edu.cn)

This work was sponsored (supported) by Joint Funds for the Innovation of Science and Technology, Fujian Province (Grant Number: 2019Y9112) and the Scientific Research Project of the National Key clinical specialty construction project (No. 22YBL-ZD-01 and No.22YBL-JB-09).

**Additional Materials:** The following are available online at ****.

Table S1: Clinicopathological parameters of sinonasal papillomas. Table S2: GSTT1 expression (Cytoplasmic expression) in sinonasal papillomas. Table S3: UCLK1 expression (Cytoplasmic expression) in sinonasal papillomas. Table S4: HLA-G expression (Cytoplasmic expression) in sinonasal papillomas. Figure S1: Methylation and sample distribution before data normalization. Figure S2: Screening of differentially methylated loci. Figure S3: Expression of GSTT1 in sinonasal papilloma (IHC, DAB, ×200). Figure S4: Expression of UCLK1 in sinonasal papilloma (IHC, DAB, ×200). Figure S5: Expression of HLA-G in sinonasal papilloma (IHC, DAB, ×200).

Table S1. Clinicopathological parameters of sinonasal papillomas

| Grade | n | Sex | | Years | Location | | |
| --- | --- | --- | --- | --- | --- | --- | --- |
|  |  | Male | Female |  | Nasal | Sinus | Others |
| normal mucosa  I | 23  20 | 17  17 | 6  3 | 46.7±17.93  53.6±10.66 | 23  17 | 2 | 1 |
| II | 63 | 47 | 16 | 53.8±11.24 | 44 | 16 | 3 |
| III | 19 | 16 | 3 | 55.9±12.00 | 11 | 5 | 3 |
| IV | 13 | 12 | 1 | 58.9±14.89 | 6 | 5 | 2 |

Table S2. GSTT1 expression (Cytoplasmic expression) in sinonasal papillomas

| Grade | n | Negative (n, %) | Weak-Expression (n, %) | Medium-Expression (n, %) | Strong-Expression (n, %) |
| --- | --- | --- | --- | --- | --- |
| normal mucosa | 23 | 0(0) | 5(21.74%) | 18(78.26%) | 0(0) |
| I | 20 | 1(5%) | 8(40%) | 11(55.00%) | 0(0) |
| II | 63 | 3(4.76%) | 35(55.56%) | 22(34.92%) | 3(4.76%) |
| III | 19 | 1(5.26%) | 10(52.63%) | 7(36.84%) | 1(5.26%) |
| IV | 13 | 0(0) | 7(53.85%) | 5(38.46%) | 1(7.69%) |

Table S3. UCLK1 expression (Cytoplasmic expression) in sinonasal papillomas

| Grade | n | Negative (n, %) | Weak-Expression (n, %) | Medium-Expression (n, %) | Strong-Expression (n, %) |
| --- | --- | --- | --- | --- | --- |
| normal mucosa | 23 | 2(8.7%) | 14(60.87%) | 7(30.43%) | 0(0) |
| I | 20 | 0(0) | 17(85%) | 3(15%) | 0(0) |
| II | 63 | 2(3.17%) | 45(71.43%) | 16(25.4%) | 0(0) |
| III | 19 | 4(21.05%) | 10(52.63%) | 4(21.05%) | 1(5.26%) |
| IV | 13 | 0(0) | 10(76.92%) | 3(23.08%) | 0(0) |

Table S4. HLA-G expression (Cytoplasmic expression) in sinonasal papillomas

| Grade | n | Negative (n, %) | Weak-Expression (n, %) | Medium-Expression (n, %) | Strong-Expression (n, %) |
| --- | --- | --- | --- | --- | --- |
| normal mucosa | 23 | 0(0) | 2(8.70%) | 17(73.91%) | 4(17.39%) |
| I | 20 | 0(0) | 0(0) | 19(95%) | 1(5%) |
| II | 63 | 0(0) | 17(26.98%) | 42(66.67%) | 4(6.35%) ^#^ |
| III | 19 | 1(5.26%) | 1(5.26%) | 11(57.89%) | 6(31.58%) ^&&^ |
| IV | 13 | 0(0) | 0(0) | 10(76.92%) | 3(23.08%) ^&^ |

#P<0.05 compared with I; &P<0.05, &&P<0.01 compared with II.


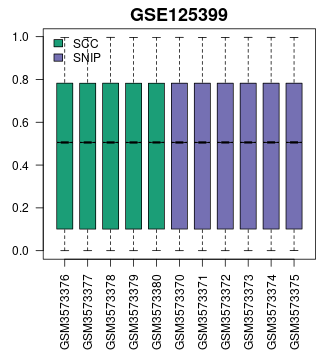

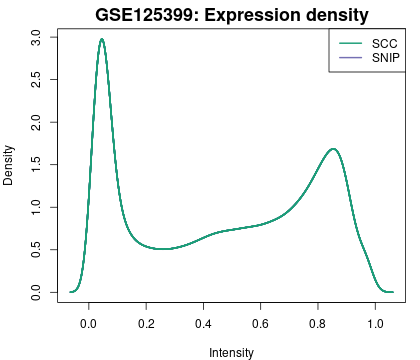


**A**

**B**

**D**


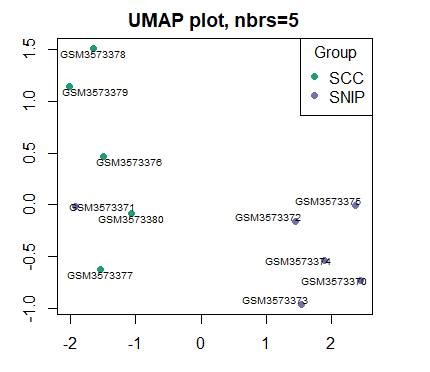

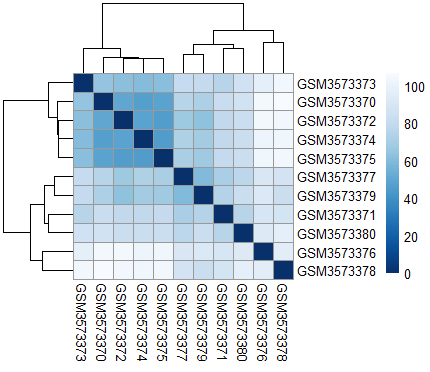


**C**

**C**

Figure S1. Methylation and sample distribution before data standardization

1. Boxplot of gene methylation after normalization. Abscissa for individual samples and ordinate for methylation signal values. Green boxed samples are SNIP-SCC group samples and purple boxed samples are SNIP group samples. B. Methylation signal value density profiles. The abscissa is the methylation signal values and the ordinate is the sample density corresponding to the size of the methylation signal value. Green curves are SNIP-SCC group samples and purple curves are SNIP group samples. C. Dimensionality reduction analysis UMAP plot. The abscissa is the relative distance. Green dots are SNIP-SCC group samples and purple dots are SNIP group samples. The distance between points represents the similarity between samples. D. Sample distance relationship matrix Heatmap. Rows with columns as individual samples. The color inside the square is proportional to the sample distance, with the closer the distance, the darker the color. Above vs. left are the phylogenetic trees that were hierarchically clustered according to sample distance, with the closer the distance, the closer the branch distance.


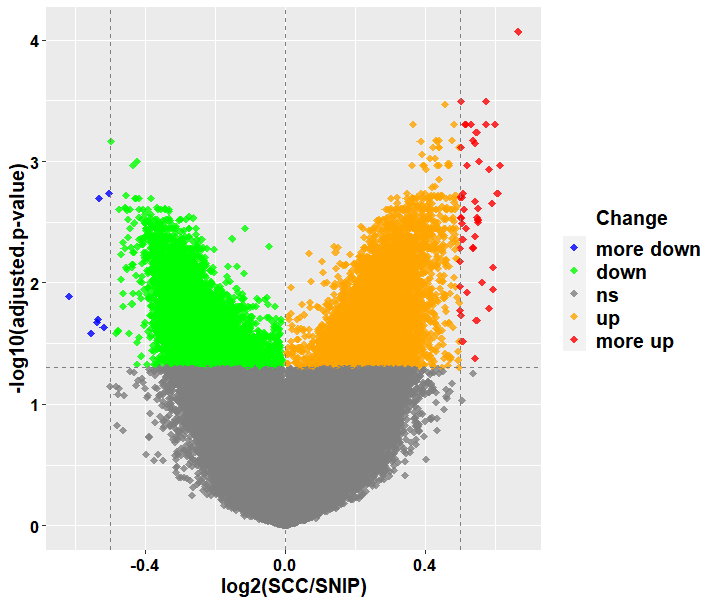


**A**


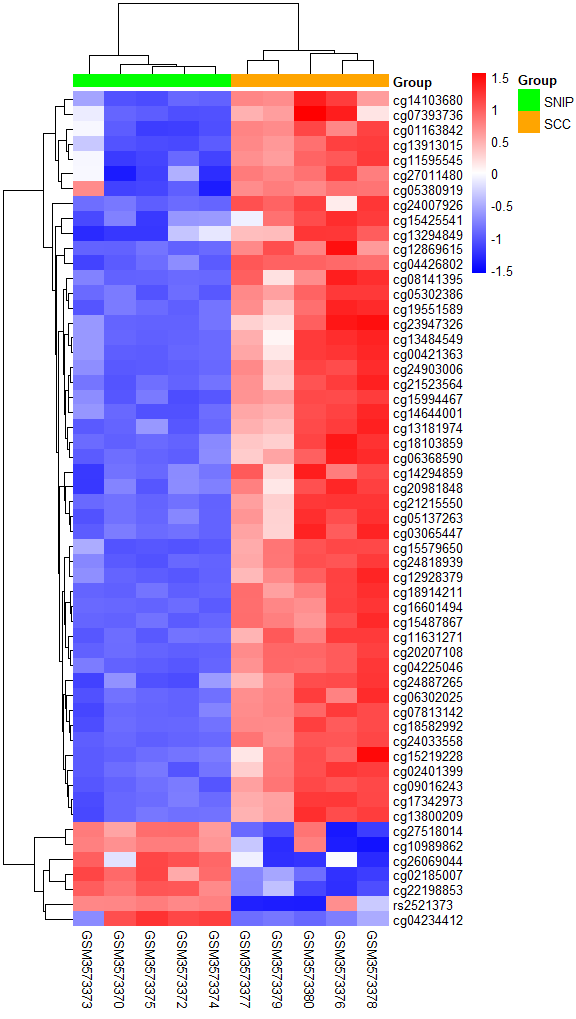


**B**

Figure S2. Screening of differentially methylated loci

A: Methylation Heatmap. The abscissa is the log2 transformed value of the ratio of the methylation signal value of the SNIP-SCC group to the SNIP group, and the ordinate is the - log10 transformed value of the corrected p value. The color of the dots represents the methylation loci that met different thresholds: (1) methylation loci with log2 (SCC / SNIP) < = - 1 and - log10 (adjusted. P-value) > 1.3 were defined as more significantly down regulated loci; (2) Methylation loci with - 1 < log2 (SCC / SNIP) < 0 and - log10 (adjusted. P-value) > 1.3 were defined as down regulated loci (down); (3) Methylation loci with 0 < log2 (SCC / SNIP) < 1 and - log10 (adjusted. P-value) > 1.3 were defined as up-regulated loci (up); (4) Methylation loci with log2 (SCC / SNIP) > = 1 and - log10 (adjusted. P-value) > 1.3 were defined as more significantly upregulated loci (more up); (5) The remainder were loci where methylation did not change (NS). B: Methylation loci Heatmap. Each column is as one sample, the green group on the left is as SNIP samples, and the orange group on the right is as SNIP-SCC samples. Each behaving one loci, the methylation signal in each sample was scaled between - 1.5 and 1.5, with positive numbers in red and negative numbers in blue, and the larger the absolute value, the darker the color. Above vs. left are hierarchical clustering system trees of samples with methylation loci, with closer distances followed by closer tree branches. On the right are the respective loci corresponding probe numbers.

| 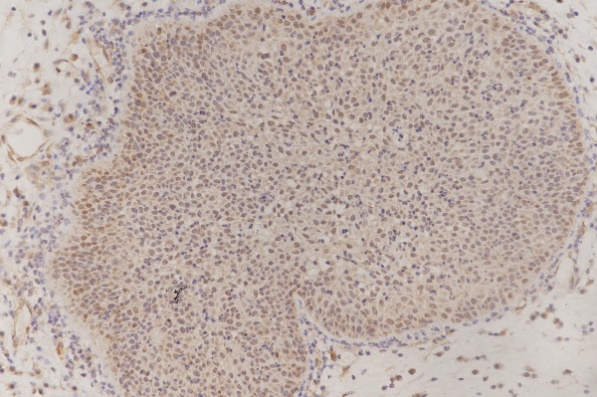**A** | **B**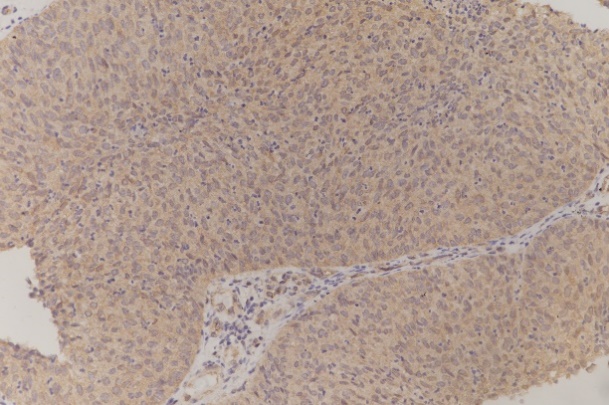 |
| --- | --- |
| 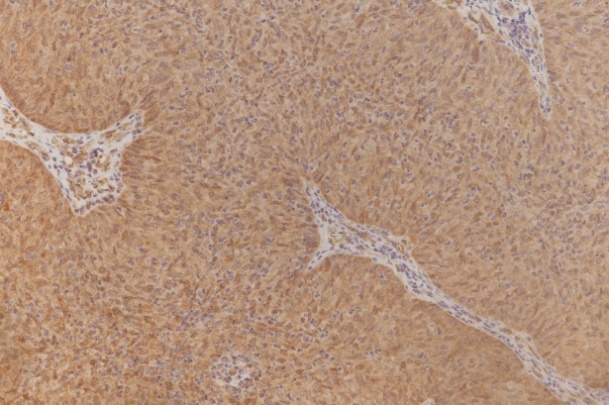**C** |  |

Figure S3. Expression of GSTT1 in sinonasal papilloma (IHC, DAB, ×200)

A: Weak expression of GSTT1 in sinonasal papilloma; B: Moderate expression of GSTT1 in sinonasal papilloma; C: Highly expression of GSTT1 in sinonasal papilloma.

| 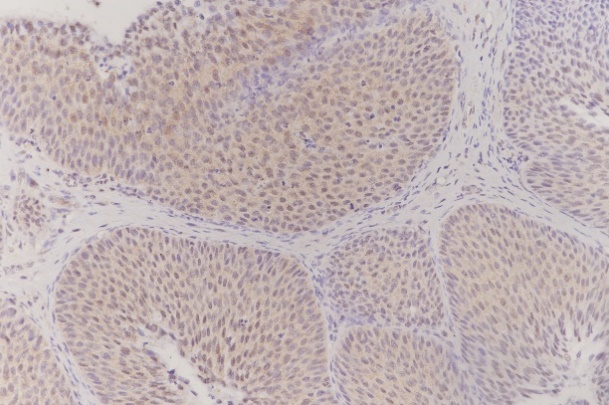**A** | 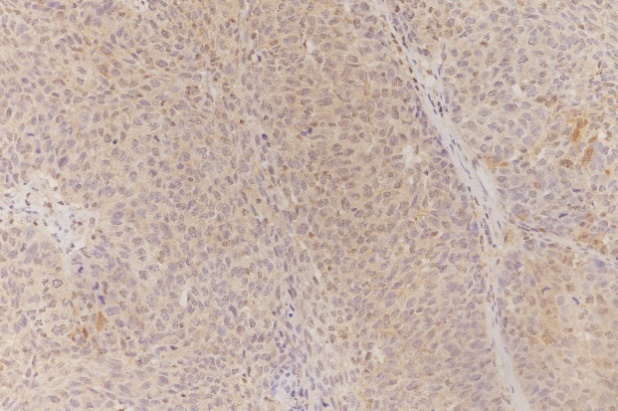**B** |
| --- | --- |
| 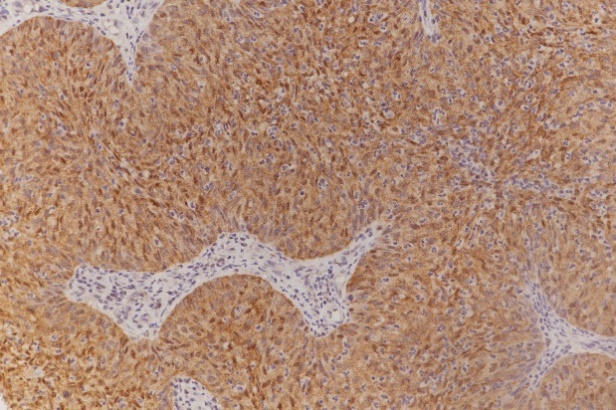**C** |  |

Figure S4. Expression of UCLK1 in sinonasal papilloma (IHC, DAB, ×200)

A: Weak expression of UCLK1 in sinonasal papilloma; B: Moderate expression of UCLK1 in sinonasal papilloma; C: Highly expression of UCLK1 in sinonasal papilloma.

| 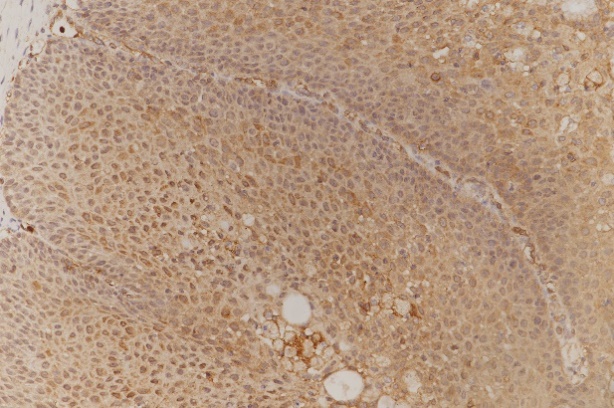**A** | 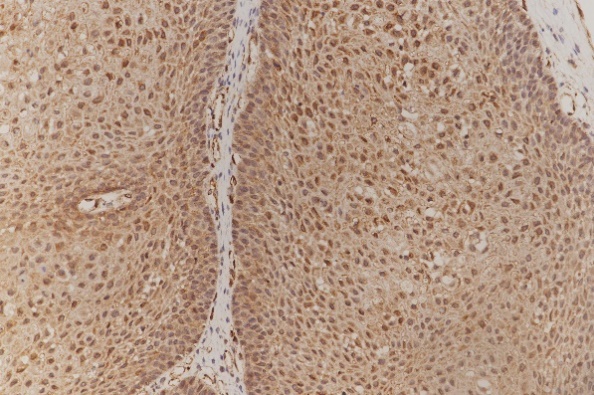**B** |
| --- | --- |
| 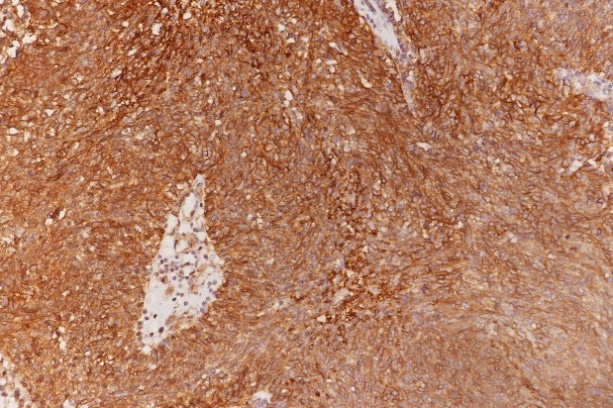**C** |  |

Figure S5. Expression of HLA-G in sinonasal papilloma (IHC, DAB, ×200)

A: Weak expression of HLA-G in sinonasal papilloma; B: Moderate expression of HLA-G in sinonasal papilloma; C: Highly expression of HLA-G in sinonasal papilloma.
